# From the archives: evolutionary origins of Delphinieae flowers, pseudogenes, and the light-responsive localization of COP1

**DOI:** 10.1093/plcell/koad312

**Published:** 2023-12-14

**Authors:** Ching Chan

**Affiliations:** Assistant Features Editor, The Plant Cell, American Society of Plant Biologists; Department of Life Science, National Taiwan Normal University, Taipei 11677, Taiwan

## March 2023: Evolutionary origins of Delphinieae flowers

**Figure 1.**
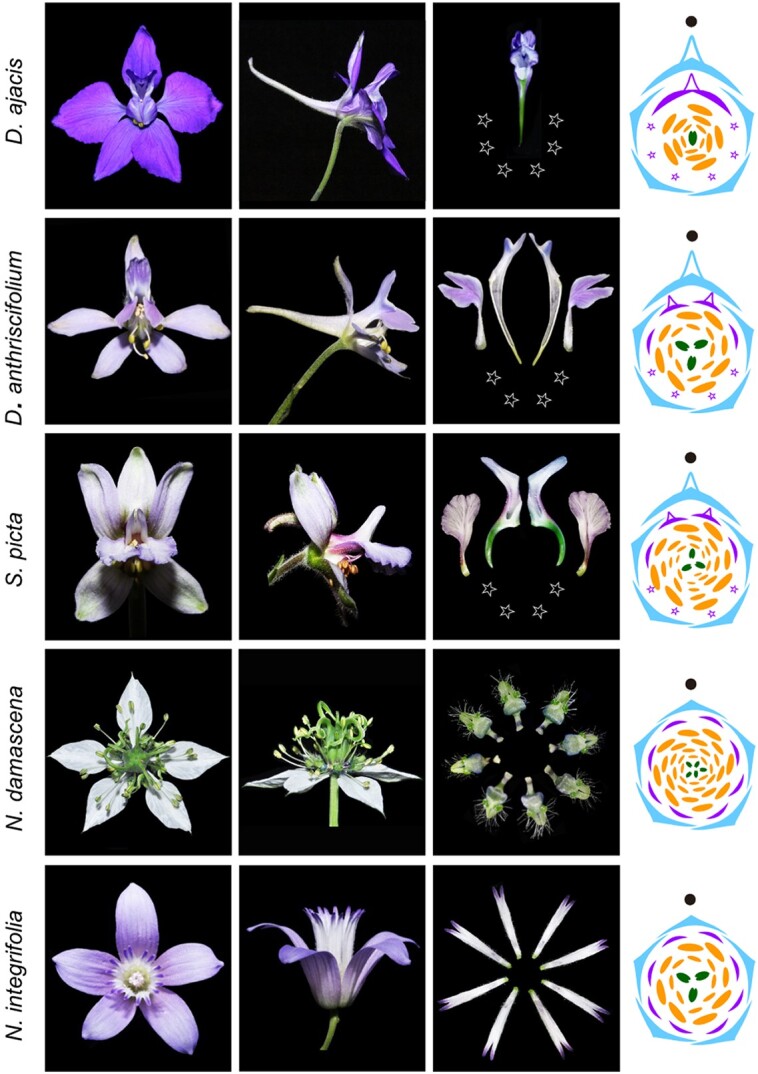
Evolution of zygomorphic flowers in Delphinieae. Three Delphinieae lineages represented by *Delphinium ajacia*, *D. anthriscifolium*, and *Staphisagria picta* (differing in perianth composition and gross morphology) and the closely related outgroups *Nigella integrifolia* and *N. damascena*. Zhao et al. (2023) show how the evolution of these floral characterisics is tied to the floral organ identity genes (*AP3* and *AGL6* lineage) and floral symmetry genes (*CYC* and *DIV* lineage). Top and side view of flowers (left) and diagrams of petals and flower of the representative species (right). Diagram color key: sky blue, sepal; purple, petal; orange, stamen; dark green, carpel; star, reduced petal. Adapted and modified from Zhao et al. (2023) Figure 1.

Floral symmetry not only holds an inherent charm and aesthetic attraction but also carries profound biological significance. Within the Ranunculaceae family, encompassing members with actinomorphic (radially symmetric) or zygomorphic (bilaterally symmetric) flowers, lies a fundamental question about the evolutionary trajectory of floral symmetry and the pivotal genes steering its diversification. In the study by Zhao et al. (2023), a comparison of transcriptomes between Delphinieae and Nigelleae species revealed intriguing insights. The investigation discovered duplication events in specific floral organ identity genes (the AP3 and AGL6 lineage) and floral symmetry genes (the CYC and DIV lineage) predating the separation of Delphinieae from other Ranunculaceae taxa (see Fig. 1). Notably, orthologs of select Delphinieae genes exhibited heightened expression in contrast to their Nigelleae counterparts. To delve deeper, the authors conducted expression knockdown experiments using virus-induced gene silencing, observing the subsequent impact on floral organ identity. Remarkably, the silencing of AP3-1 and -3, AGL6-1a, and CYC2a resulted in the formation of less actinomorphic flowers. The once-spurred sepals transitioned to a nonspurred state, resembling the outgroup Nigelleae lineage. Thus, the intricate interplay between floral organ identity genes and floral symmetry genes stands as the driving force behind the diverse floral landscape of the Delphinieae within the Ranunculaceae family.

## March 2019: Evolutionary origins of pseudogenes

Pseudogenes, nonfunctional replicas of protein-coding genes, often emerge from genome duplication or integration events, displaying various degenerative traits such as frameshifts, in-frame stop codons, and truncated sequences within full-length genes (Zhang et al. 2003). To facilitate the identification of pseudogenes, Xie et al. (2019) introduced an innovative computational tool, PlantPseudo, which annotated around 250,000 pseudogenes across 7 Angiosperm species. Surprisingly, an average of 23% of plant genes possessed pseudo-counterparts, yet with minimal overlap between species. Intriguingly, the sequence alterations within these pseudogenes, including insertions, deletions, and stop codons, demonstrated nonrandom patterns, exhibiting stronger selection at the 5′ and 3′ termini. A significant portion of these pseudogenes exhibited proximity to their functional protein-coding counterparts, implying potential functional associations. Through a re-examination of published chromatin immunoprecipitation sequencing data, the authors observed an enrichment of histone marks linked to the expression of long noncoding RNAs (lncRNAs) within the terminal regions of these pseudogenes. Subsequent transient expression experiments substantiated the activity of these cis-regulatory elements in driving the expression of fluorescent reporter proteins. Therefore, despite lacking the ability to encode proteins, pseudogenes may generate RNA molecules that potentially contribute to post-transcriptional regulatory mechanisms.

## March 1999: Light-responsive localization of COP1

The CONSTITUTIVELY PHOTOMORPHOGENIC 1 (COP1)/SUPPRESSOR OF PHYA-105 (SPA) E3 ubiquitin ligase plays a pivotal role in repressing photomorphogenesis. Activation of light-sensitive photoreceptors triggers the exclusion of COP1 from the nucleus, thereby disrupting the COP1-SPA interaction. Consequently, this disruption leads to the deactivation of the E3 ubiquitin ligase function, enabling the stabilization of factors promoting photomorphogenesis (Podolec and Ulm 2018). While the molecular mechanism behind COP1-mediated photomorphogenic response is now well established, the specific signal responsible for mediating COP1 movement between the nucleus and cytoplasm was not known 25 years ago. Stacey et al. (1999) noted several conserved motifs within the N-terminal of the COP1 protein. Through the use of truncation constructs, the authors identified a bipartite nuclear localization signal (NLS) within a central “core” domain and a cytoplasmic localization signal (CLS) embedded in the RING domain. Interestingly, neither the NLS nor the CLS alone can independently govern light-dependent nucleo-cytoplasmic movement. Only upon reconstitution of the N-terminal region, housing both the core and RING domains, did light-regulated nuclear localization become evident. Hence, it became apparent that the collaborative interaction between the 2 signals—rather than either the NLS or CLS alone—confers light responsiveness.

## Dive Curated Terms

The following phenotypic, genotypic, and functional terms are of significance to the work described in this paper:

CLS Gramene: AT4G04870
CLS Araport: AT4G04870

COP1 Gramene: AT2G32950
COP1 Araport: AT2G32950

AGL6 Gramene: AT2G45650
AGL6 Araport: AT2G45650

AP3 Gramene: AT3G54340
AP3 Araport: AT3G54340
